# Do You Hear the Same? Cardiorespiratory Responses between Mothers and Infants during Tonal and Atonal Music

**DOI:** 10.1371/journal.pone.0106920

**Published:** 2014-09-10

**Authors:** Martine Van Puyvelde, Gerrit Loots, Pol Vanfleteren, Joris Meys, David Simcock, Nathalie Pattyn

**Affiliations:** 1 Research Group Interpersonal, Discursive and Narrative Studies (IDNS), Faculty of Psychology and Educational Sciences, Vrije Universiteit Brussel (VUB), Brussels, Belgium; 2 VIPER Research Unit, Royal Military Academy (RMA), Brussels, Belgium; 3 Universidad Católica Boliviana “San Pablo”, La Paz (UCB), Bolivia; 4 Department of Mathematical Modeling, Statistics and Bio informatics, Faculty of Bioscience Engineering, University of Ghent (UG), Ghent, Belgium; 5 Institute of Food, Nutrition and Human Health, Massey University, Palmerston North, New Zealand; 6 Faculty of Medicine and Bioscience, James Cook University, Queensland, Australia; 7 Department of Experimental and Applied Psychology, Vrije Universiteit Brussel (VUB), Brussels, Belgium; Max Planck Institute for Human Cognitive and Brain Sciences, Germany

## Abstract

This study examined the effects of tonal and atonal music on respiratory sinus arrhythmia (RSA) in 40 mothers and their 3-month-old infants. The tonal music fragment was composed using the structure of a harmonic series that corresponds with the pitch ratio characteristics of mother–infant vocal dialogues. The atonal fragment did not correspond with a tonal structure. Mother–infant ECG and respiration were registered along with simultaneous video recordings. RR-interval, respiration rate, and RSA were calculated. RSA was corrected for any confounding respiratory and motor activities. The results showed that the infants’ and the mothers’ RSA-responses to the tonal and atonal music differed. The infants showed significantly higher RSA-levels during the tonal fragment than during the atonal fragment and baseline, suggesting increased vagal activity during tonal music. The mothers showed RSA-responses that were equal to their infants only when the infants were lying close to their bodies and when they heard the difference between the two fragments, preferring the tonal above the atonal fragment. The results are discussed with regard to music-related topics, psychophysiological integration and mother-infant vocal interaction processes.

## Introduction

Since the time of the Ancient Greeks, there has been debate regarding whether the preference for consonance over dissonance has cultural or biological origins. Consonance refers to the aesthetic evaluation of multiple tones that sound harmonious, stable or pleasant, whereas dissonance refers to tones that sound unharmonious and unpleasant (e.g., [1]). Several studies on adults have reported a preference for consonance or simple frequency ratios above dissonance or complex frequency ratios [2]–[5]. In studies of central nervous system activity, listening to dissonant music has been associated with heightened activity in areas of the brain that are known to be involved in stress responses [2], [4], [6], [7]. For instance, listeners show increased activity in the amygdala, hippocampus [4] and parahippocampal gyrus [2], [8] during dissonant music and decreased activity in these regions during joyful consonant music [4]. It has been suggested that the parahippocampal gyrus might be involved in the processing of affective vocal signals [6]. In the amygdala, it seems that different nuclei are involved in the responses to consonance and dissonance [6]. For instance, activity in the superior regions is related to dissonance [9], whereas activity in the basolateral [9] and lateral [6] regions is related to consonance. Pallesen et al. [7] stated that neural responses can be evoked even with isolated chords and that such responses do not differ between musicians and non-musicians.

To understand the origins of the preference for consonance, infant studies have used the looking-time preference procedure to demonstrate a preference for consonance in infants at the ages of 2 days [10], 2 months [11], 4 months [11], [12] and 6 months [13]. Masataka’s [10] study of 2-day-old infants was exceptional because prenatal experience was eliminated by including hearing infants of deaf parents. This study concluded that infants prefer consonance over dissonance from birth onwards, independent of prenatal or early postnatal experience. By contrast, a recent study [14] that utilized the same looking-time preference procedure did not observe a preference for consonance in 6-month-old infants and, thus, did not support an innate preference for consonance. Considering these inconsistent findings, the use of other determinants to infer musical preferences is instructive. In a recent study [15], adult participants reported that their musical preference was related to their physiological responses such as the sensation of chills and shivers, feelings of bodily tension or relaxation and so forth.

In a number of infant studies, respiratory sinus arrhythmia (RSA) has been utilized as a measure of physiological response because it can be obtained through non-invasive ECG-respiratory registration (e.g., [16]). RSA provides an accurate measure of a component of the natural heart rate variability (HRV) that is present during a respiration cycle as a result of regulation by the parasympathetic division of the autonomic nervous system via the vagus nerve [17]. Heart rate (HR) accelerates during inspiration and decelerates during expiration. Thus, the degree of RSA provides an indication of the vagal control of the heart. That is, as vagal tone increases, the relationship between HRV and the respiratory cycle becomes more pronounced and RSA increases [17]. From an integrated psychophysiological point of view, RSA is one of the responses that are related to arousal-induced responses in social situations [18]. It has been speculated that during low demands for social engagement, vagal tone increases and HR decreases, allowing the body to focus on internal processes. By contrast, during challenging situations, vagal tone decreases and HR increases to prepare for environmental participation and self-regulation [19].

Most infant studies that have examined the physiological responses to music have focused on the clinical context of music therapy with preterm infants. The results of these studies are inconsistent, likely due to the differences in preterm stage and/or differences in type of musical stimulation used across studies. Most of the studies have reported that music therapy has soothing and stabilizing effects on preterm infants [20] and that these effects are often related to decreased HR (e.g., [21], [22]). However, one study that utilized maternal voice and singing [23] observed increased infant HR in response to both maternal speech and maternal singing with no significant difference in HR response between the two stimulations [23]. Furthermore, a study that combined Kangaroo Care with live harp music therapy [24] did not observe a physiological impact of the music on the infants’ HR. Finally, a developmental study [25] observed decreased HR in response to three different types of musical stimuli among infants at 3 and 6 months of age, but increased HR at 9 months of age and no effect at 12 months of age. To our knowledge, no studies on infants’ physiological responses to consonance and dissonance have been conducted.

In adult studies, music-related physiological responses appear to be inconsistent. In an early study, increased HR and arousal were reported in response to preferred, relaxing music [26]. By contrast, studies in a nursing context [27]–[29] have found the opposite result. Krumhansl [30] observed that sad music induced HR-decrease and, Sammler, Grigutsch, Fritz and Koelsch [31] reported dissonance-related decreased HR. However, Gomez and Danuser [32] did not find a significant variation in HR in response to music. When examining HRV, a few studies [33]–[35] have reported increased parasympathetic activity in response to relaxing, sedative music. Furthermore, music that is self-rated as strongly emotional and/or inducing chills is related to increased physiological arousal [36], [37]. These results were supported by a recent study that controlled for the respiratory component of RSA and showed decreased parasympathetic activity during excitative music in comparison with sedative music [33]. As with the physiological variations that were observed in the infant studies, this inconsistency is likely due to the type of musical stimulation that the different studies employ. Moreover, caution is needed when inferring emotional interpretations or preferences from physiological events, as these events are multi-dimensional and, thus, can be affected by a variety of other processes [38]. With respect to HR, fluctuations may be the result of small changes in motor activity that are not relevant to the task performance [39], [40]. Regarding HRV, in addition to vagal outflow, RSA reflects respiratory and metabolic demand related to somatic activity [38]. RSA is negatively correlated with motor activity in both adults (e.g., [41]) and infants [42], [43]. There is also a negative relationship between RSA and respiration rate and a positive relationship between RSA and tidal volume (e.g., [41], [44]–[46]). Therefore, motor and respiratory effects must be controlled or treated as confounding factors when attempting to study psychological influences on RSA [38].

**Figure 1:**
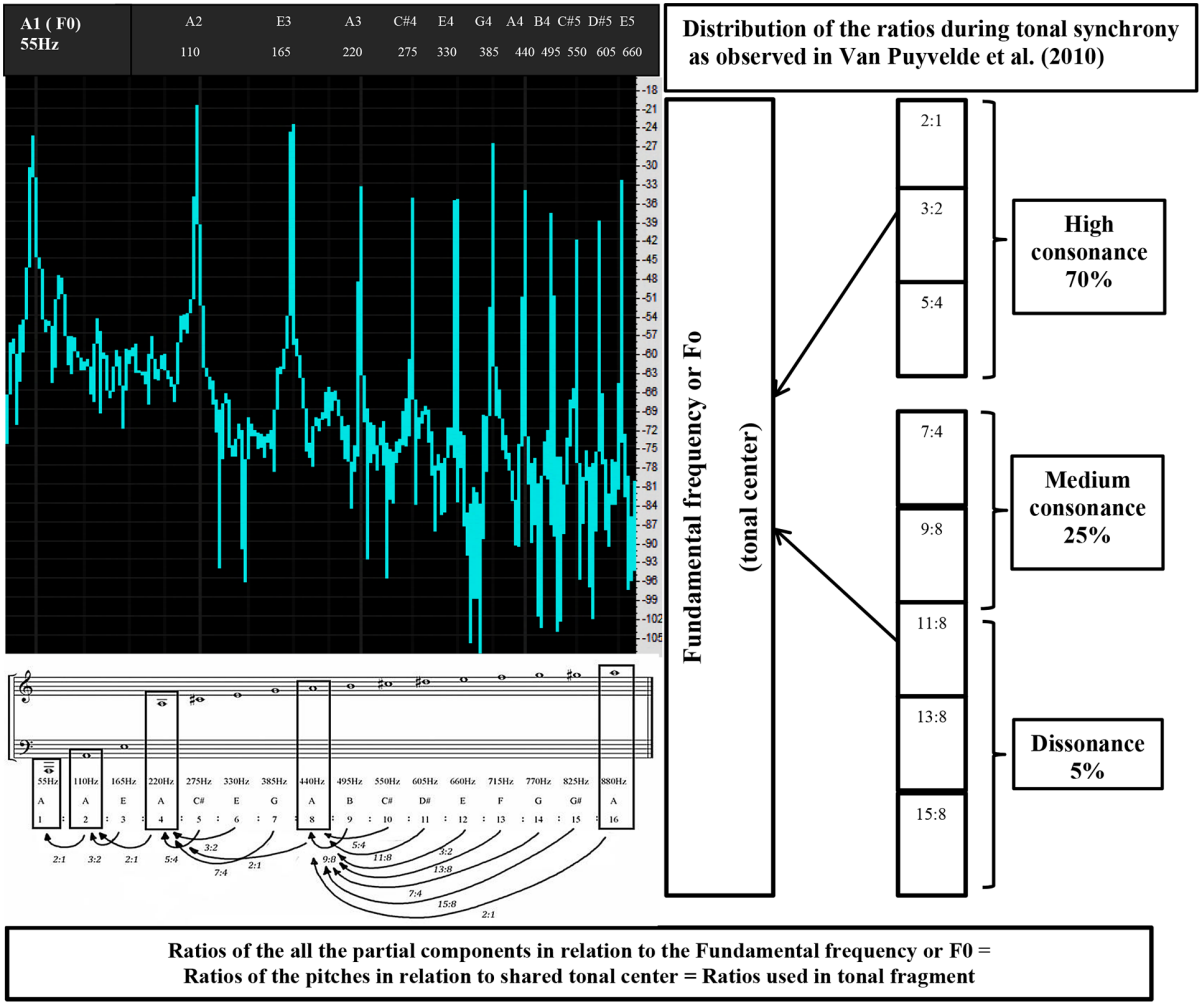
Illustration of a spectral analysis (multiple peak sounds within one tone) of a 55 Hz pitch tone. A regular sound vibration is a complex tone that consists of a fundamental frequency (F0 or harmonic number 1, h1 or A1 on the Figure, i.e., most left peak) + a series of harmonics or partials (h2–16, other peaks). Together, they blend, giving the impression of one single tone. The frequency of each partial is a multiple of the F0 (55–110–165 Hz and so forth…). These multiple relations are expressed in ratios on the Figure. For example h3 (165 Hz) is related to h2 (110 Hz) with a ratio of 3∶2. When considering the harmonics h2–16 in relation to the F0, you obtain the illustrated frequency ratios (see on the bottom of the Figure). Van Puyvelde et al. (2010) observed that, during tonal synchrony, mothers and infants share a tonal center (which corresponds with F0/h1 and its octaviations) to which their other uttered pitches are related in the same fashion as the partials of a harmonic series are related to the fundamental frequency or F0 (i.e., the mothers and infants use the same ratios as pictured on the Figure). The partials of the tonal center are indicated by rectangles and the arrows show the frequency ratios with regard to this tonal center. For example h6 is related to h4 (ratio 6∶4 = 3∶2) and not h5 because h5 is not part of the tonal center (no octaviation of F0). On the right, an overview of the distribution of the ratios during tonal synchrony and their approximated music interval is given, with a distinction between high consonance (2∶1, octave; 3∶2, perfect 5; 5∶4 major 3) medium consonance (7∶4, minor 7; 9∶8, major 2) and dissonance (11∶8, triton; 13∶8, minor 6; 15∶8 major 7). The same ratios and distribution were used in the tonal fragment, i.e., 70% high, 25% medium consonance and 5% dissonance).

In the current study, we examined the RSA-responses of 40 mothers and infants to a tonal and an atonal music fragment. Some studies have hypothesized that adult musical preferences are rooted in early mother-infant interaction processes, arguing that maternal singing and talking has an emotion regulating effect [8], [15]. For instance, Mitterschiffthaler et al. [8] associated preferences for consonance over dissonance with the universal repertoire of mothers’ lullabies, which feature simple frequency ratios [47]. Indeed, Shenfield, Trehub and Nakata [48] demonstrated that infants with high baseline levels of cortisol benefitted from maternal singing, as indicated through a decrease in cortisol. Moreover, recent studies on maternal-infant speech have shown that during mother-infant bonding moments of affect repair, the observed interrelated pitch frequency ratios of the vocalizations are tonally related or synchronized and mainly contain consonant simple frequency ratios [49], [50]. Van Puyvelde et al. [49] suggested that the association of consonant frequency ratios in maternal speech with enhanced social connectedness may be related to later consonant preference. The distribution of frequency ratios during moments of tonal synchrony appears to be in agreement with the hierarchy of a harmonic series. A harmonic series is an acoustical phenomenon. Each pitch of a regular sound corresponds with a periodic sound that consists of a harmonic spectrum with a pattern of partial components (or so-called harmonics). These harmonics have fixed ratios in relation to their fundamental frequency. Together, they blend or harmonize with this fundamental frequency and give the impression of a single tone (see Figure 1 for an illustrated explanation of a harmonic series). During tonal synchrony, mothers and infants share a tonal center to which their other uttered pitches are related in the same way as the harmonics are related to their fundamental frequency. Moreover, the observed ratios during tonally synchronized vocal dialogues are mainly consonant or simple frequency ratios (i.e., 70% of the time), with a smaller number of complex frequency ratios (i.e., 25% medium consonance and 5% dissonance) [49], [50]. A similar hierarchical response pattern has been found in the phase-locked neural activity within individuals’ brainstems while listening to different consonant and dissonant intervals [51].

The tonal music fragment that was used in the current study was based on a harmonic series and the previously described features of tonally synchronized mother-infant vocal dialogues whereas the atonal fragment was not based on a harmonic series (see method). Because tonal synchrony is a relational concept, we explored both mothers’ and infants’ responses to the music and potential coherences between the two. Therefore, the mothers and their infants listened to the music together. In this way, the current approach differed from a conservative experiment that measures tonal and atonal infant preferences but controls for the potential influence of the mother (e.g., mother is deaf to the music). We attempted to answer the following three research questions: (1) Do mothers and infants show a different RSA-response to the tonal versus atonal fragment? (2) Do mothers and/or infants show a different RSA-response to the tonal versus atonal fragment when having close body contact compared to being separated? (3) Does the mothers’ ability to differentiate between the two types of stimuli influence the RSA-responses of mothers and/or infants?

## Method

### Participants and characteristics

The study was approved by the local ethics committee (UZ-Jette, Belgium, B.U.N. 143201111237). We recruited 45 mothers from prenatal classes and a private midwife’s office. The mothers who agreed to participate were contacted a few weeks before the estimated date of their infant’s birth and signed written informed consent forms. At the time of the first recording session, the mothers’ mean age was 30 years and 3 months (SD = 3.84; range 25–43 years). All of the infants (21 boys, 19 girls) were healthy, full-term born infants and passed the Universal Newborn Hearing Screening (UNHS) test. The mean birth weight was 3.380 kg (SD = 0.376; range 2.680–4.160 kg), and the mean birth length was 49.84 cm (SD = 1.47; range 48–52 cm). The mothers’ mean duration of higher education was 6 years (SD = 2.0, range 2–10 years). The recordings of 40 dyads were used for the current analyses; 5 of the recordings were excluded due to artifacts (*N* = 3) or infant fussiness (*N* = 2).

### Apparatus

For the ECG and respiration registration, the ambulatory BioRadio 150 system (Cleveland Medical Devices Inc., Cleveland, Ohio, USA) was used. This system consisted of a BioRadio User Unit and Computer Unit. The User Unit had a wireless data acquisition system that allowed the subjects to move freely and acquired the synchronized ECG and breathing signals of both the mother and the infant. Digitized signals were wirelessly transmitted by the User Unit to the Computer Unit which was connected to a PC computer via USB port and later imported into the VivoSense software version 2.4 (Vivonoetics, San Diego, USA) for further analysis. Simultaneous digital video recordings were made with a DCR-SX73 E Sony Handycam (SCA, California, USA). The music fragments were composed and arranged by means of Finale, Music Notation Software Products for Music Composition (MakeMusic Inc., Minnesota, USA) and the digitalized sound samples by means of Roland XP-80 (Roland Corporation, Shizuoka, Japan). The statistical analyses were conducted using R version 3.02 (R Foundation for Statistical Computing, Vienna, Austria), with package lme4 used for the mixed effect modeling [52] and the phia package utilized for the evaluation of the interactions [53].

### Recording of physiological signals

Two standard single-channel ECG registrations (II derivation) were used, one for the mother and one for the infant. The electrodes were placed –in correspondence with the configuration of Einthoven, Fahr and de Waart [54]- on the upper right side and the lower left side of the chest. The grounding electrode was placed on the mother’s back. The grounding of the infant was obtained by maintaining the infant’s skin-to-skin contact with the mother (see procedure). The ECG signals were recorded with a sampling frequency of 960 Hz and breathing signals with a sampling frequency of 60 Hz. To register breathing movements, both the mother and the infant wore a thoraco-abdominal respiratory effort belt, with the infant wearing a pediatric belt.

### Procedure

**Figure 2:**
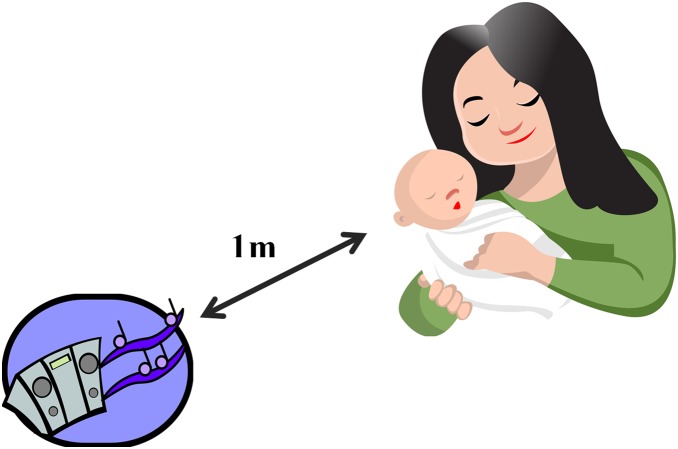
Mothers and infants listened to two music fragments. One group of the mothers maintained close body contact with their infants. The other mothers were asked to take a seated position next to the infant without close body contact. The music stereo installation was placed 1 meter from the participants. The music stimulus was played at 60 db.

The data were collected during home visits. The stereo Hifi-system was placed one meter away from the mother and the infant (see Figure 2). After fitting the electrodes and respiratory effort belts, one group of the mothers (*N* = 21) was asked to take a seated resting position with the infant lying close to the mother’s body (see Figure 2). The other mothers (*N* = 19) were asked to take a seated position next to the infant without close body contact. During registration, the mothers were asked to hold their infants’ hand or foot during the testing to ensure continuous skin-to-skin contact between the mother and the infant. When the mother felt comfortable, a 3-minute baseline registration was recorded and, subsequently, the testing commenced. The test consisted of two blocks of music, each with a duration of 3 min 12 s with a pause of 1 min 48 s between the two blocks. Before starting, the experimenter informed the mothers that they would hear two different music fragments and that they were expected to listen carefully to these fragments to later report which fragment they preferred. To provide a further motivational incentive for the mothers to pay attention to the music fragment, they were also asked to report their eventual thoughts or feelings and any notable behavioral responses that they observed from their infant during the two music fragments. In addition, the mothers were instructed not to interact with their infants during the music fragments unless they thought that their infants were in discomfort. When the infant sought explicit contact with the mother or smiled at her, the mother was allowed to respond briefly to avoid a still-face situation. Between the fragments, the mothers were allowed to talk quietly with the infants without stimulating or arousing them. The presentation order of the two music fragments was counterbalanced (tonal-atonal, *N* = 19; atonal-tonal, *N* = 21).

### Music fragments

**Figure 3:**
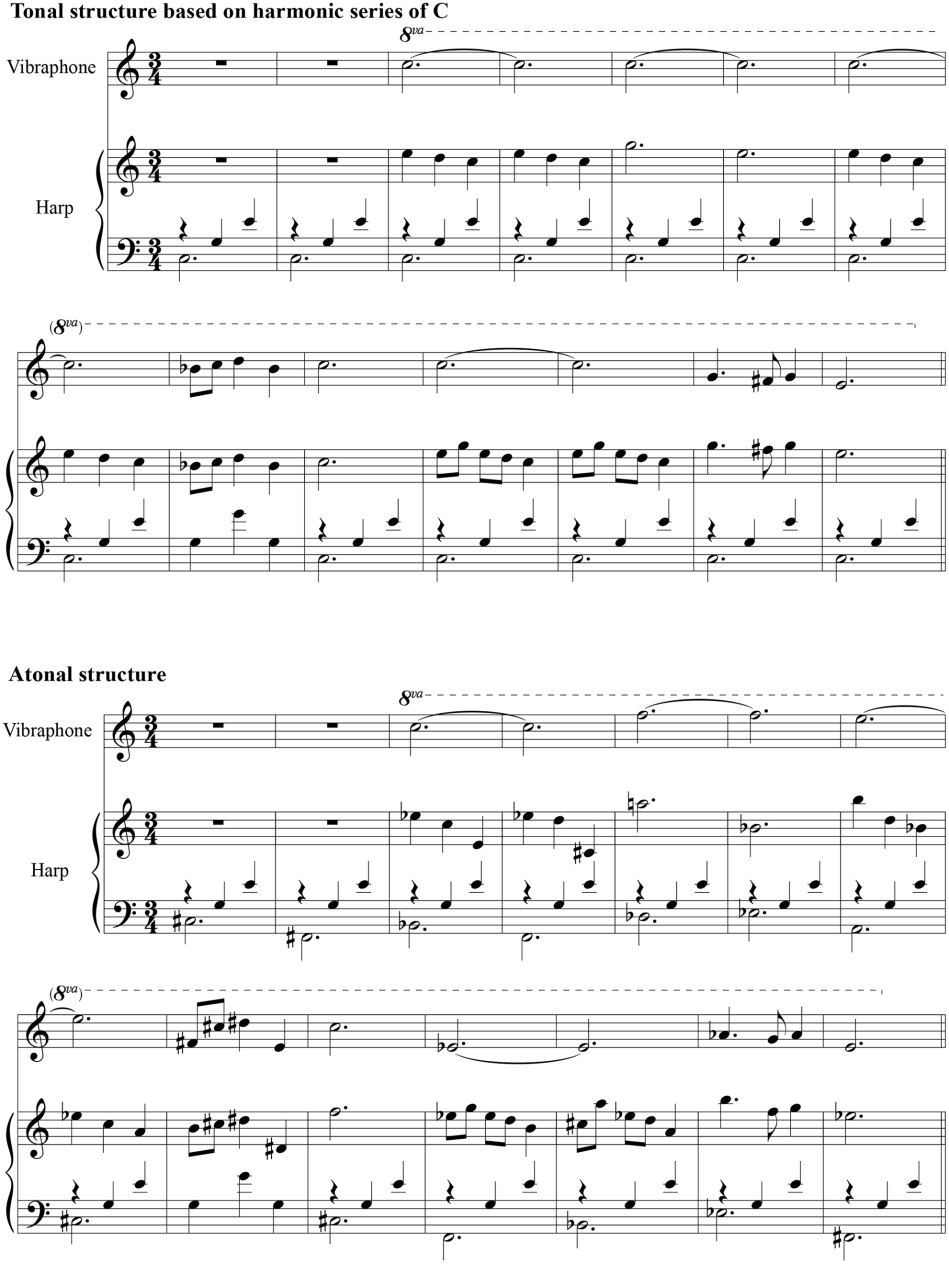
Illustration of the first 14 bars of the two music fragments to show the tonal structure and the disruption of tonality. Fragment 1 (Tonal structure) is composed with the notes of the harmonic series C, i.e., C-G-E-Bb-D-F#. The ratios in relation to the tonal center C consisted of 70% high consonance (i.e., the notes C-E-G), 25% medium consonance (i.e., the notes Bb, D) and 5% dissonance (i.e., the notes, F#, G#, B). In Fragment 2 (Atonal structure), the harmonic series musical structure was absent.

Fragment 1 (tonal) was based on the notes of the harmonic series of C. Thus, the composition consisted of only 8 notes (plus the octaviations). These notes corresponded with the first 16 harmonics or partials of the complex tone C, i.e., C-G-E-Bb-D-F#-G#-B. Moreover, the incidence of simple and complex frequency ratios aligned with the harmonic series and tonal synchrony, i.e., 70% high consonance (C-E-G), 25% medium consonance (Bb, D) and 5% dissonance (F#, G#, B). In Fragment 2 (atonal structure), the harmonic series musical structure was absent. To minimize habituation, every 10 s, the leading musical instrument and rhythms of music were varied to attain the infant’s attention with next sounds of Roland XP-80 (Roland Corporation U.S., Los Angeles, USA), a music workstation expanded with the SR-JV80-02 and SR-JV80-09 Wave Expansion Boards: harp, vibraphone, piano, and pizzicato strings. This variation and other musical determinants, such as length, tempo, volume (i.e., 60 db), rhythms and instrument sound samples, were identical in the two fragments (see Figure 3).

### Analysis of physiological signals

#### Data analysis

For the analyses, we computed maternal and infant RR-interval (RRI), respiratory sinus arrhythmia (RSA) and respiration frequency (fR) during all testing blocks. These analyses of the recorded signals were performed using proprietary algorithms in the dedicated VivoSense software (Vivonoetics, San Diego, USA), which achieves R-wave detection and RSA-calculation through a derivative-based algorithm that accounts for violations of the Nyquist-criterion (see next paragraph). The timing of the detected R-wave was used to generate the RR-interval (RRI). For each testing block, maternal and infant fR, RRI and RSA were calculated. RSA was computed using the peak-valley method (i.e., the mean difference between the shortest heart period associated with inspiration and the longest heart period associated with expiration for each respiratory cycle) to reflect vagal tone [55]. This is the most appropriate method for infant research, as it accounts for Nyquist-violations (see next paragraph). Frequency-domain spectral analysis requires a minimum of at least two minutes of uninterrupted registration to generate stable RSA-estimates [43], [56]. All of the ECG and breathing data were visually inspected for artifacts and incorrect detections. In the case of ectopic beats or erroneous detections, the data were manually corrected with the removal of the erroneous detection/artifact followed by a cubic spline interpolation (corrections were applied to <1% of data).

The need to correct RSA for respiration is even more important in infant studies than in adult studies (see [43]). Young infants possess an immature control of their respiration [57] and a high respiration rate [56] with small breathing amplitude [58], which can complicate the RSA calculation. When the respiration rate is overly rapid to allow the detection of two succeeding RRI-periods during one inspiration or expiration, the Nyquist-criterion (i.e., the requirement that the sampling rate is at least twice as high as the frequency of interest) is violated [56], [59]. VivoSense accounts for violations of the Nyquist-criterion and scores the breaths with no detectable peak-valley RSA as zero. To address the potential effects of respiration frequency on RSA, we conducted a series of within-subject regressions on the averages of the data of each experimental block, predicting infants’ RSA from its respiration frequency. This approach is consistent with a previously published methodology [60]. Residuals from these regressions were collected and used to control for the effect of respiration frequency on RSA [41], [60]. Although non-invasive ambulatory respiratory inductance plethysmography has been adapted to infants and used in previous studies [43], [61], we were not in the possession of such a device. Therefore, we did not include a measurement of tidal volume in the present study. An additional common issue in psychophysiological research is the large between-individual variation in RRI, RSA and fR measures. Hence, within-subjects z-transformations were applied, as recommended in a previously published methodology (e.g., [62]).

### Data preparation

#### Influence of maternal and infant motor behavior

In agreement with recent methodology [42], [43], two independent coders analyzed the video-recordings second-by-second for motor behavior [42], [43]. The coding scheme of Bazhenova et al. [42] was created to code infant motor activity. Therefore, we adapted it to the mother’s motor activity (i.e., 0 = quiet motor, 1 = slow, 2 = mild/moderate, 3 = pronounced). In line with Ritz et al. [43], the percentage of time that an infant or a mother spent in each activity level was calculated and then multiplied by the activity level value. Then, the values were summed, resulting in a range from 0 (maximum of ‘quiet motor’) to 300 (maximum of ‘pronounced’). The inter-rater reliability between the two independent coders was assessed on a random 25% of the recordings. During coding, both of the coders were blind to the test condition. High kappa inter-rater reliabilities were reached, *M = *.82 (Cohen’s κ).

#### Additional cognitive and physical variables

Eleven mothers did not hear the difference between the two fragments. Therefore, we included ‘differentiation’ as a variable in the analysis model. Furthermore, 21 mothers listened to the fragments with the (dressed) infant close to their bodies, whereas the other 19 mothers listened without close body contact with their infants. As previously mentioned, in both groups, the mothers maintained skin-to-skin contact by holding their infants’ foot or hand. All of the mothers who perceived a difference between the tonal and atonal fragment preferred the tonal fragment.

### Statistical analysis

To answer the current research questions, we fit separate coefficients for mothers and infants; these coefficients took into account all of the aforementioned factors. For each observation, the variable “Subject” indicated whether the measurement originated from the mother or the infant. The variable “Condition” referred to the baseline, the tonal fragment or the atonal fragment. For both variables, treatment contrasts were used, with “Mother” or “Baseline” as the reference value, respectively. The two other indicator variables were “Together” and “Differentiation”, which evaluated the effect of close body contact between the mother and the infant and the mothers’ ability to differentiate between the two fragments, respectively. The research questions were tested within the framework of Linear Mixed Effect models. Every model was fitted using restricted maximum likelihood. A random intercept was fitted for every individual, and the model was corrected for variability in motor activity during the testing. The model was selected using a backwards approach, starting from the model with all possible 2-, 3- and 4-way interactions. In each step, all n-way interactions were dropped, starting with the 4-way interaction. The reduced model was then compared with the model from the previous step using a likelihood ratio test. This procedure ensures that the models were suited to post-hoc testing of the interactions. After selecting the final model, post-hoc comparisons were used to answer the research questions. To evaluate the interactions, an approach that was similar to Boik’s [63] approach was chosen. P-values were corrected for multiple testing using the method of Holm [64].

## Results

### Overview

**Figure 4:**
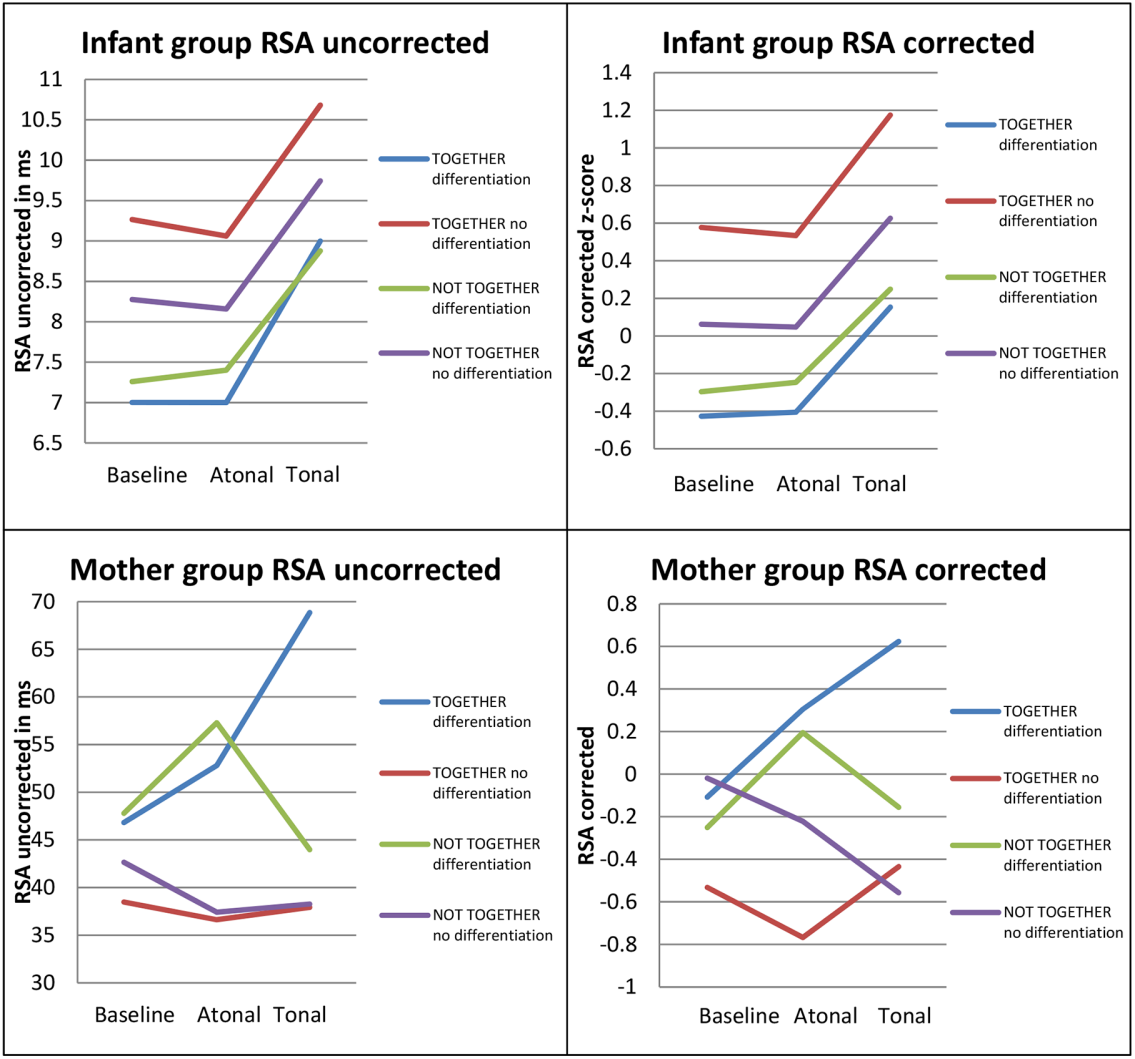
Overview of RSA-responses before and after correction for respiratory and motor confounds in mother group and infant group.

Figure 4 provides an overview of the RSA-responses to the tonal and atonal fragment in the mother group and infant group before and after the correction of RSA for the confounding effects of motor and respiratory activities.

### Selected model

The final model included all of the described variables and interactions, apart from the 4-way interaction. The removal of the 3-way interactions resulted in a significant loss of explained variance, as shown by the likelihood ratio tests, *p* = .014 (see Table 1).

**Table 1:** Output of the likelihood ratio test comparing the model with and the model without the 3-way interaction. AIC, BIC and log likelihood are given for each model.

Model	df	AIC	BIC	Log Likelihood	deviance	Chi sq	Chi df	*p*
**3 way interactions**	18	526.09	588.74	−245.04	490.09			
**2 way interactions**	23	521.93	601.99	−237.97	475.93	14.15	5	.0147*

*Note.* Signifcant *p-*values are indicated in bold font with an asterisk (*).

### Research question 1: Do mothers and infants show a different RSA-response to the tonal versus atonal fragment?

Table 2 shows that the infants’ and the mothers’ RSA-responses to the tonal and atonal music differed. The infants showed significantly increased RSA-levels when listening to the tonal fragment compared to both the atonal fragment (χ^2^(1) = 21.78, *p*<.001) and baseline (χ^2^(1) = 23.78, *p*<.001). The mothers did not seem to react to the different music fragments, exhibiting no significant differences in their RSA-responses to the three different conditions (see Table 2).

**Table 2:** Post hoc comparisons for the mothers and infants for the effect of the tonal/atonal music on the corrected RSA.

Subject	Music fragment	Value	df	Chi sq	*p*
**Mother**	**baseline-atonal**	−0.18	1	2.28	0.5251
	**baseline-tonal**	−0.10	1	0.74	1.0000
	**atonal-tonal**	0.07	1	0.42	1.0000
**Infant**	**baseline-atonal**	−0.02	1	0.02	1.0000
	**baseline-tonal**	−0.57	1	23.78	<.0001*
	**atonal-tonal**	−0.55	1	21.78	<.0001*

The value represents the difference in RSA for the groups.

*Note.* Signifcant *p-*values are indicated in bold font with an asterisk (*).

### Research question 2: Do mothers and/or infants show a different RSA-response to the tonal versus atonal fragment when having close body contact compared to being separated?

Body contact between the mothers and infants impacted the RSA-responses. Table 3 shows that there was a significant difference in RSA-responses between mothers and infants when they were not in close body contact. This was the case both when the mother differentiated between the two fragments, χ^2^(2) = 10.20, *p* = .018, and when there was no differentiation between fragments, χ^2^(2) = 13.97, *p* = .004 (see Table 3).

**Table 3:** Differences between the infants and mothers within every group.

Comparison	Body contact	Differentiation Mother	Condition 1	Condition 2	df	Chi Sq	*p*
**Mother-infant**	**Together**	**Differentiation**	0.15	−0.24	2	3.78	.3016
		**No differentiation**	−0.50	−0.31	2	2.34	.3103
	**Apart**	**Differentiation**	−0.45	−0.85	2	10.20	.0183*
		**No differentiation**	−1.10	−0.91	2	13.97	.0037*

Condition 1 and Condition 2 are fitted coefficients representing the effect of the variable Condition.

*Note.* Signifcant *p-*values are indicated in bold font with an asterisk (*).

### Research question 3: Does the mothers’ ability to differentiate between the two types of stimuli influence the RSA-responses of mothers and/or infants?

The mothers’ ability to differentiate between the tonal and atonal fragment only had a significant effect on the mothers’ responses. There was a significant difference in RSA between mothers who differentiated and mothers who did not differentiate, χ^2^(2) = 9.70, *p* = .016. Within the infant group, there was no significant difference in RSA between the infants whose mothers differentiated fragments and the infants whose mothers did not χ^2^(2) = 0.13, *p* = .94. The results of the post-hoc test are provided in Table 4.

**Table 4:** Post hoc test for the effect of the differentiation capacity on the response differences for both the infant and the mother.

Comparison	Subject	Condition 1	Condition 2	df	Chi Sq	*p*
**Differentiation-No differentiation**	**Mother**	−0.63	0.02	2	9.70	.0156*
	**Infant**	0.02	0.08	2	0.13	.9351

Condition 1 and Condition 2 are fitted coefficients representing the effect of the variable Condition.

*Note.* Signifcant *p-*values are indicated in bold with an asterisk (*).

### Conclusion tested by post-hoc comparisons for dyads with close body contact and with mothers who differentiated between the two fragments

**Figure 5:**
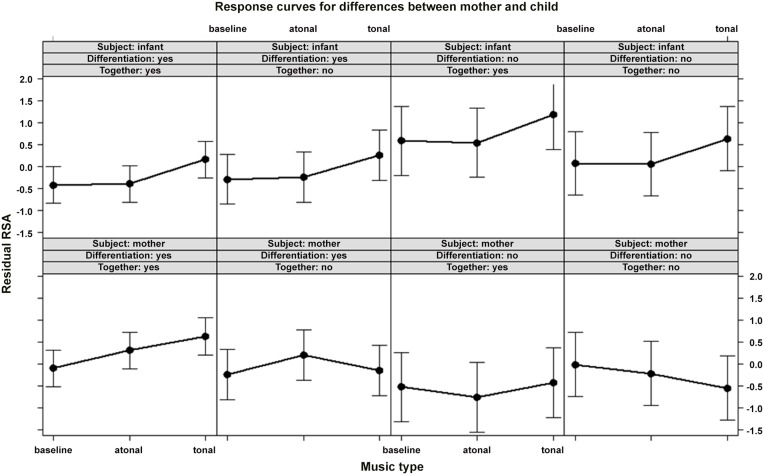
Overview of the RSA-responses of mothers and infants in each group.

Research question 2 showed that there was no difference in the response between mothers and infants during close body contact regardless of the mothers’ ability to differentiate the two music fragments. Nevertheless, the ability to differentiate the fragments had a significant effect on the mothers’ but not the infants’ responses (research question 3). Therefore, post-hoc comparisons of the different listening conditions were conducted in the mother group and infant group for the four possible combinations (i.e., 1/close body contact–differentiation, 2/close body contact – no differentiation, 3/no close body contact – differentiation, and 4/no close body contact – no differentiation). These comparisons revealed that mothers and infants only showed similar RSA-responses to tonal music when the two were in close contact and the mother differentiated the two fragments. In such cases, both the mother and the infant showed increased RSA-levels during the tonal fragment in comparison with baseline levels (see Table 5). The infants also showed a significant difference in RSA-response to the tonal versus atonal fragment, χ^2^(1) = 14.91, *p* = .0025. The comparisons between the different listening conditions in the other groups were not significant (*p*>.05) (see Table 5). A detailed overview of the mothers’ and infants’ RSA-responses for each group is shown in Figure 5.

**Table 5:** Overview of the effect on RSA of listening to the tonal and atonal fragments for infants and mothers.

Subject	Condition	Music fragment	Value	*df*	*Chi sq*	*p*
**Mother**	**Together/Differentiation (** ***N*** ** = 19)**	Baseline-atonal	−0.41	1	8.27	.0804
		baseline-tonal	−0.73	1	25.16	<.0001*
		atonal-tonal	−0.32	1	4.82	.3378
	**Together/No Differentiation (** ***N*** ** = 5)**	baseline-atonal	0.23	1	1.04	1.000
		baseline-tonal	−0.10	1	0.18	1.000
		atonal-tonal	−0.33	1	2.06	1.000
	**Not Together/Differentiation (** ***N*** ** = 10)**	baseline-atonal	−0.44	1	5.63	0.229
		baseline-tonal	−0.10	1	0.26	1.000
		atonal-tonal	0.35	1	3.49	0.678
	**Not Together/No Differentiation (** ***N*** ** = 6)**	baseline-atonal	0.20	1	0.83	1.000
		baseline-tonal	0.54	1	5.88	0.214
		atonal-tonal	0.34	1	2.28	1.000
**Infant**	**Together/Differentiation (** ***N*** ** = 19)**	baseline-atonal	−0.02	1	0.02	1.0000
		baseline-tonal	−0.58	1	16.20	.0013*
		atonal-tonal	−0.56	1	14.91	.0025*
	**Together/No Differentiation (** ***N*** ** = 5)**	baseline-atonal	0.04	1	0.04	1.000
		baseline-tonal	−0.60	1	6.66	0.161
		atonal-tonal	−0.64	1	7.69	0.106
	**Not Together/Differentiation (** ***N*** ** = 10)**	baseline-atonal	−0.05	1	0.07	1.000
		baseline-tonal	−0.55	1	8.46	0.076
		atonal-tonal	−0.50	1	7.00	0.147
	**Not Together/No Differentiation (** ***N*** ** = 6)**	baseline-atonal	0.02	1	0.01	1.000
		baseline-tonal	−0.56	1	6.35	0.176
		atonal-tonal	−0.58	1	6.737	0.161

Value indicates the difference between the groups.

*Note.* Signifcant *p-*values are indicated in bold with an asterisk (*).

## Discussion

The present study examined the effects of two music fragments (i.e., tonal or atonal) on the RSA-responses of 40 mothers and their 3-month-old infants. The two music fragments differed from one another only in the presence (tonal) or absence (atonal) of a harmonic series musical structure. The tonal fragment was intended to correspond with the tonal structure that was observed during tonally synchronized mother-infant vocal dialogues, whereas the atonal fragment was intended to correspond with non-tonally synchronized vocal dialogues [50]. In the analyses, motor activity was included as a covariate and RSA was controlled for effects of respiration rate. This discussion addresses music-related and maternal-infant interaction-related topics.

The findings with regard to research question 1 showed that the infants’ and the mothers’ RSA-responses to the tonal and atonal music differed. Whereas mothers’ RSA did not seem to react to the music in general, the infants showed significantly higher RSA-levels during the tonal fragment than during the atonal fragment and baseline. We first discuss the results of the infants’ responses to tonal versus atonal music with regard to a potential consonance bias. In the literature, there is a controversy with regard to the presence [10]–[13] or absence [14] of a biological predisposition to consonance bias. In the present study, the infants showed increased RSA-levels during the tonal fragment in relation to baseline levels and in relation to the atonal fragment. This effect was observed independent of the varying RSA-responses of the mothers over the music conditions, which are discussed below. Physiological reactivity has been designated as a reliable determinant of musical preference in adult studies [15], suggesting that infants preferred the tonal and overly consonant music to the atonal dissonant music. However, musical preferences in adult studies are related to arousal (e.g., [65]) and chills [36], [37]) rather than to relaxing aspects. In a context of infant development, physiological relaxation may be preferred over physiological arousal to obtain self-regulation (e.g., [66]–[68]). However, such conclusions are beyond the scope of the current study. We can only conclude that the infants showed a differentiation in RSA-responses between the tonal and the atonal fragment. It is possible that the music fragments constituted a significant environmental stimulus that overrode other possible environmental stimuli, including entrainment to the mother’s RSA when in physical contact. A hypothesis in the literature is that consonant preferences may originate from early experiences during mother-infant vocal dialogues as a consequence of the regulating impact of melodious mother talk and mother singing [8], [15], [49]. The tonal fragment in the present study was based on these tonal features of early vocal dialogues between mothers and infants. Therefore, the overall RSA-responses of the infants during music based on these characteristics of vocal interaction warrant further study into whether the tonal qualities of early vocal dialogues are related to successful affective [49] and physiological co-regulation.

We did not observe an effect of atonal music in comparison to the baseline on the infants’ RSA-responses even though a decrease in RSA during the atonal music fragment might have been expected. It is possible that the effect of the atonal music fragment was reduced as a result of the continuous touch between the mothers and infants during the music conditions. A recent research [69] showed that touch between a mother and infant attenuates the infant’s physiological reactivity to stress during a still-face experiment (i.e., a simulated situation of maternal deprivation in which the mother is not allowed to respond to the infant). Without their mothers’ touch, the infants showed increased cortisol levels and decreased RSA during the still-face phase and did not recover during reunion. However, when mothers were allowed to touch their infants throughout the experiment, the magnitude of the stress response in RSA and cortisol was reduced and the infants recovered during reunion [69].

In comparison with previous studies of infants’ responses, the current findings correspond with the reported soothing effects of music therapy on preterm infants’ HR (e.g., [20]–[22]). However, the present results are in contrast to other reports of arousal in preterm infants during maternal talk and singing [23] and during musical stimuli [25]. One explanation for these inconsistencies may be differences in the nature of the musical stimuli (i.e., maternal voice, orchestral pieces, harp, piano, tempo, rhythm and so forth) that were utilized. With regard to tempo differences, it has been suggested that the observed cardiovascular responses of adults may be influenced by musical and instrumental expression [70] or entrainment processes [71]. Due to entrainment, decreases or increases in HR in response to music may occur when the tempo of the music is much lower or higher than the mean HR (i.e., 80 beats per minute in adults). However, in the present study, it is likely that the music-related factors did not influence the results, as the tempo, instrumentation and intensity were identical in the two fragments. Another reason for the inconsistencies across the studies may relate to the population that was tested. Most of the reported studies were conducted with preterm infants who tend to be easily stimulated [20] in comparison to full-term infants (i.e., the present study sample).

This study included the mothers’ responses in the analyses. Some mothers did not differentiate between the two music fragments. The mothers who differentiated between the fragments, preferred the tonal above the atonal fragment. It was notable that the mothers displayed RSA-responses that were equal to those of their infants only when they differentiated between the two fragments and when the infants were lying close to their bodies. By contrast, all of the infants responded to the tonal fragment regardless of their mothers’ responses. These results may suggest that the music fragments provided a more significant stimulus to physiological response in the infants than in the mothers. Moreover, it appears that physiological reactivity to music in adults is a top-down regulated process, whereas infants at this age seem to respond via bottom-up processes. In the mothers, we likely observed an integrated mind-body response to the music, as described in the neurovisceral integration models [18], [72]–[73], resulting in the mixed response pattern. This notion is in line with some studies’ suggestion that the human musical inner life is a culturally developmental [74] and age-dependent process (e.g., [74]–[75]) that is related to cognitive functions such as meaningfulness, needs and beliefs that are developed over the life-time (e.g., [15]). It has yet to be determined how and at what age top-down processes begin to interfere with physiological responses to music.

The mothers’ and infants’ responses in this study may be supported by animal study results that full body contact is required to achieve maternal-infant matched physiology [76]. In human adults, an additional cognitive component may be needed. This suggestion is supported by a study that showed that physiological synchrony between mothers and infants, only occurred when mothers were able to respond in a sensitive manner [77]. However, the current findings and suggested interpretations contrast with other studies that reported physiological synchrony between mothers and infants who had no bodily contact [78]–[79]. It has been proposed that different underlying mechanisms are responsible for eliciting physiological synchrony when body contact is or is not present [78], but variations in methodology may explain the apparent variability between studies. Until now, mother-infant physiological synchrony was studied in social interactive situations that required movement and speech, which are known to elevate the metabolism (e.g., [43], [60], [80], [81]) and induce respiration variability [43]. These potential confounds were statistically controlled in the current study. Therefore, further replication studies in the domain of mother-infant synchrony that account for motor and respiratory confounds are warranted to allow more reliable comparisons between studies.

This study had several merits and limitations. In contrast to most RSA-studies that have been conducted in a mother-infant context, we controlled for respiration and motor activity. However, we did not control for tidal volume and, thus, did not analyze the potential influences of volume-related variability. An additional limitation was the ambiguity as to why some mothers did not differentiate the two music fragments. We did not test the mothers’ ability to process melody and harmony and, therefore, do not know whether these mothers were tone-deaf or not focused on the music. Although this issue has no significance with regard to the current interpretations, further research may be advised to include a music perception test on tone-deafness (e.g., the Montreal Amusia Test Battery [82]). One of the most significant contributions of the current study was the relational approach that was used to explore the RSA-responses of mothers and infants when exposed to tonal versus atonal music. However, because this study is the first to explore RSA-responses to music in a mother-infant context, the findings must be further elaborated. For instance, the infants’ RSA-responses to the two music fragments and the differentiation between the body contact conditions may be weakened by the fact that the mother and infant maintained some body contact during the recordings to maintain instrument grounding [69]. Secondly, additional research is warranted to reveal the potential one-sided or two-sided (non)conscious influences between the mothers and infant that contributed to the current findings. Finally, an analysis of mothers’ and infants’ emotional responses to music can offer an additional perspective.

In summary, the present study demonstrated that the infants showed increased RSA-levels to a tonal fragment of music that had the characteristics of tonally synchronized mother-infant vocal dialogues in comparison to a baseline and an atonal fragment. The mothers responded with similar increased RSA-levels to a tonal fragment only when they were holding their infant close to their body and when they heard the difference between the two fragments, with a preference for the tonal fragment.
